# An Accessible Approach to Molecular and Biomarker Testing for Risk Stratification in Oral Cavity Squamous Cell Carcinoma

**DOI:** 10.1007/s12105-026-01968-2

**Published:** 2026-09-18

**Authors:** Michelle Afkhami, N. Haghighi, H. Ma, A. Reyes, F. Fei, T. Dyer, M. Telatar, J. Arias-Romero, Y. Haghighi, A. Mehrazma, E. Massarelli, R. Bakkar, P. Tizro, D. Bell, V. M. Villaflor

**Affiliations:** 1 Department of Pathology, Division of Molecular Pathology & Therapy Biomarkers CLIA Director, COH-Clinical Molecular Genomics and Cytogenomics Laboratories Medical Director, Cytogenetics and Clinical Molecular Diagnostic Laboratories, City of Hope Comprehensive Cancer Center Chief, 4900 Rivergrade, Suite D220-A2 Room 2138, 91706 Irwindale, CA USA; 2 Department of Computational and Quantitative Medicine-Beckman Research Institute, City of Hope Comprehensive Cancer Center, Duarte, CA USA; 3 Department of Medical Oncology, City of Hope Comprehensive Cancer Center, Duarte, CA USA; 4 Department of Internal Medicine, Division of Oncology and Hematology, University of Texas Tyler, Tyler, TX USA; 5 Department of Pathology, Division of Anatomic Pathology, City of Hope Comprehensive Cancer Center, Duarte, CA USA; 6 Department of Pathology, University of Pittsburgh Medical Center, Pittsburgh, PA USA; 7 Chao Family Comprehensive Cancer Center, Department of Medical Oncology, Division of Head and Neck Oncology, University of California Irvine, Orange County, CA USA

## Abstract

**Introduction:**

Oral cavity squamous cell carcinoma (OCSCC) is a common epithelial malignancy for which site-specific biomarkers with proven prognostic utility remain limited. In this study, we present one of the largest single-center, site-specific molecular analyses of OCSCC to date. Using two separate versions of multigene next-generation sequencing (NGS), we sought to identify a focused set of clinically actionable and widely accessible biomarkers that could support diagnosis, prognostication, and therapeutic decision-making. Our findings highlight the potential of a limited molecular panel to capture key prognostic information, supporting a more accessible and scalable approach to precision oncology in OCSCC.

**Patients and Methods:**

Seventy-three adult patients (ages 19–85 years) with site-specific OCSCC treated at City of Hope between 2019 and 2023 were included. Tissue specimens were collected at initial diagnosis or recurrence and analyzed using NGS panels for both DNA and RNA. Sequencing identified single nucleotide variants, copy number alterations, and gene fusions. Associations between genomic alterations and overall survival (OS) were assessed using Kaplan–Meier curves and log-rank tests with significance set at *p* < 0.05.

**Results:**

*TP53* and *TERT*-promoter oncogenic variants were the most frequent alterations, co-occurring in 64.4% of cases regardless of disease stage (primary vs. recurrence). Pathogenic variants in *CDKN2A*,* HRAS*,* PIK3CA*,* BRCA1*, and *CASP8* were enriched among patients who developed recurrence. In this group, copy number amplification of *CCND1*,* FGF3*,* FGF4*,* FGF19*,* RPS6KA4*,* RPS6KB2*, and *MYC*, along with copy number loss of *CDKN2A* and *CDKN2B*, were observed. Lower PD-L1 expression, absence of *TERT*-promoter mutations (PMs), and focal amplification of 11q13 were associated with poor overall survival in univariate analysis (all *p* < 0.05).

**Conclusions:**

Multigene NGS profiling revealed recurrent genomic alterations and prognostic biomarkers in OCSCC. Based on these findings, we propose an initial biomarker panel comprising TP53 and PD-L1 (22C3 clone) immunostains, along with TP53 and TERT PM analysis. Collectively, these assays capture key prognostic biomarkers associated with adverse outcomes, including low PD-L1 expression (CPS < 20) and the absence of TERT PMs in relation to overall survival. In addition, copy-number assessment of 11q13, MYC (8q), and CDKN2A (9p) may warrant further investigation for future clinical application.

**Supplementary Information:**

The online version contains supplementary material available at https://doi.org/10.1007/s12105-026-01968-2.

## Introduction

Head and neck squamous cell carcinomas (HNSCCs), exhibit considerable heterogeneity in histology, etiology, prognosis, and genomic landscapes, with these distinctions heavily influenced by anatomic sites. In the United States, an estimated 59,660 new cases of oral cavity and pharyngeal cancer were projected as of 2025, representing approximately 2.9% of all new cancer diagnoses, with about 12,770 deaths expected from oral cavity or oropharyngeal cancer [1]. Within this group, oral cavity squamous cell carcinoma (OCSCC) is one of the most common epithelial neoplasms of the mouth. According to the National Cancer Institute’s Surveillance, Epidemiology, and End Results (SEER) database, OCSCCs are defined as tumors arising from the lip, hard and soft palate, retromolar trigone, anterior two-thirds of the tongue (mobile tongue), gingiva, buccal mucosa, and floor of the mouth [1].

Traditionally, OCSCC has been diagnosed through surgical pathology and immunohistochemistry (IHC), with p16 and PD-L1 IHCs serving as key markers for prognostic and therapeutic stratification. Despite decades of genomic research, no definite site-specific biomarker signature has been established for OCSCC.

Multigene molecular profiling has identified several key drivers of tumorigenesis and clinical behavior across HNSCCs, including OCSCC [2–4]. Among these, *TP53* mutations are the most frequently observed, accompanied by recurrent chromosomal alterations such as gains in 3q, 7p, 8q, 11q, and 20q, and losses in 3p, 8p, 9p, and 18q [3–5]. Clinically, adverse prognostic indicators, including a history of smoking, higher pathological T-stage, and *TP53* mutation status, are strongly associated with increased risk of extranodal extension (ENE) and poorer clinical outcomes [6].

While prior studies have advanced the molecular understanding of HNSCCs, most have relied on pooled data from heterogeneous subsites and included HPV-positive tumors, limiting site-specific insights for OCSCC. Consequently, despite its high incidence, there remains a lack of robust prognostic and predictive biomarkers for this disease, hindering personalized treatment. Additionally, current risk stratification primarily relies on clinicopathological features and tumor staging [7], yet patients with similar profiles often experience varying outcomes.

To address these gaps, we conducted a retrospective analysis of clinical, pathological, biomarker, and multigene molecular data from a distinct OCSCC cohort.

## Methods

### Study Cohort and Data Collection

We identified 388 adult patients with HNSCC treated at City of Hope (COH) between January 2019 and December 2023. Of these, 97 cases were classified as OCSCC. Seventy-three patients were longitudinally followed for a median follow-up of 45.5 months to assess subsequent clinical outcomes. Inclusion was restricted to tumors arising from anatomic sites defined by the SEER database as part of the oral cavity, with exclusion of other head and neck subsites.

This study was approved by the COH Institutional Review Board and conducted in accordance with institutional ethical and consent guidelines (IRB #15198). Demographic and clinicopathologic variables, including age at diagnosis, alcohol consumption based on amount and frequency, smoking history, HPV status, American Joint Committee on Cancer (AJCC) stage (I–IV), surgical margin status, extranodal extension (ENE), perineural invasion, and tumor differentiation (well, moderately, or poorly differentiated) were collected through the Precision Oncology Software Environment Interoperable Data Ontologies Network (POSEIDON) platform at COH.

### Genomic Profiling

Genomic analysis was performed on tumor specimens from 73 patients, including 38 primary tumors and 44 recurrent tumors; 9 patients contributed both primary and recurrent specimens.

Among these, 51 specimens were sequenced using whole-exome sequencing (WES) or the HopeSeq Solid Tumor Comprehensive (HSC) assay including DNA sequencing of 523 genes for mutations, copy number changes in 500 genes, microsatellite instability (MSI), tumor mutation burden (TMB), and RNA sequencing of 165 genes, including both known and novel gene fusions. While remaining samples were analyzed using the HopeSeq Solid Tumor Complete (HS) panel with about 165 genes for DNA sequencing and 51 genes for RNA sequencing. All analysis were performed at COH Clinical Molecular Diagnostics CLIA and CAP approved laboratories. Analyses focused on the subset of genes shared across all sequencing platforms. A detailed list of panels and genes included in each assay was provided in Supplementary Tables 1 A and 1B.

### Copy Number Alteration Analysis

Copy number status was determined using validated, vendor-established copy number variation (CNV) cutoff values for each gene. Copy number loss (CNL) was defined as a fold-change value < 0.6 in samples with tumor cellularity > 20%. Copy number amplification was defined as a fold-change value > 1.6 with a minimum tumor cellularity of 20%.

### Variant Calling

Only pathogenic and likely pathogenic variants, including both somatic mutations and CNVs, were included in the analysis. Variants were curated and classified according to the AMP/ASCO/CAP tier classification guidelines. Variants were required to meet a minimum allelic frequency threshold of 5% and a minimum sequencing depth of 250×. The average sequencing coverage per case was ≥ 1800 reads.

### Microsatellite Instability

Microsatellite instability (MSI) was calculated as the ratio of unstable MSI sites to the total number of assessed MSI sites. Tumors were classified as MSI-high (≥ 20%) or MSI-stable (< 20%).

### Tumor Mutational Burden

Tumor mutational burden (TMB) was defined as the number of non-synonymous somatic single nucleotide variants (SNVs) and small insertions/deletions (indels) per megabase (mut/Mb) within coding regions, using the human genome build 38 references. TMB was categorized as high (> 17 mut/Mb), intermediate (> 5 and ≤ 17 mut/Mb), or low (≤ 5 mut/Mb).

### PD-L1 Immunohistochemistry

PD-L1 immunohistochemistry (IHC) using the 22C3 clone and combined positive score (CPS) interpretation was performed uniformly on 67 cases. While PD-L1 expression is generally defined as CPS ≥ 1 in solid tumors, for OCSCC, high PD-L1 expression is defined as CPS ≥ 20, and low expression as CPS < 20, in accordance with prior studies [8, 9].

### P16 Immunohistochemistry

HPV status was inferred from p16 immunohistochemistry because molecular HPV testing was not performed uniformly across the cohort. Based on available records, p16 was negative in all cases.

### Statistical Analysis

Demographic and pathologic characteristics were summarized using standard descriptive statistics. Comparisons of categorical variables were performed using chi-square tests or Fisher’s exact tests, as appropriate. Associations between demographic and pathologic variables and overall survival (OS) were assessed using Kaplan–Meier survival curves and log-rank tests in univariate analyses. OS was defined as the time from diagnosis to death from any cause and was censored at the date of the most recent follow-up visit, for patients who were alive. Disease-free survival (DFS) was similarly evaluated using Kaplan–Meier methods and log-rank tests. DFS was defined as the time from diagnosis to the first occurrence of recurrence, metastasis, death from any cause, or last follow-up. Recurrence was defined as the return of cancer after a period of no evidence of disease (NED) and was classified as local (primary site), regional (adjacent tissues or lymph nodes), or distant (metastatic disease). For this study, sites at or above the clavicle (excluding the brain) were considered locoregional, while sites below the clavicle were classified as distant recurrence or metastasis. All statistical tests were two-sided, with a significance defined as *p* < 0.05. Analyses were performed using SAS version 9.4 (SAS Institute, Cary, NC).

## Results

### Demographics/Clinicopathology

Seventy-three patients with a confirmed diagnosis of OCSCC were included in this Single-institution retrospective cohort study with longitudinal outcome follow-up. The median age at diagnosis was 64 years (range, 19–85), with a slight female predominance (54.8%) (Table 1A). Among patients who reported the corresponding demographic characteristics, the majority were Caucasian (71.4%). A history of cigarette smoking was reported in 29 patients (40.3%). Regarding alcohol consumption, 45 patients (61.6%) reported occasional intake, while 11 patients (15.1%) reported moderate to heavy consumption.

Of the 73 patients, 48 underwent surgery followed by adjuvant chemoradiation, while 15 received neoadjuvant chemotherapy with either cisplatin, 5-fluorouracil, and docetaxel (TPF) or carboplatin/paclitaxel, followed by surgery and adjuvant chemoradiation. Eight patients underwent surgery alone. Of the remaining two patients, one had metastatic disease and died before receiving treatment, while the other declined additional therapy after biopsy. Therefore, all tumors included in this study were immunotherapy-naïve at the time of sequencing.

Among patients with available clinicopathology characteristics, the oral tongue and floor of mouth were the predominant primary sites of OCSCC, accounting for 57.5% of cases (Table 1). Histologically, the majority of tumors were moderately differentiated (*n* = 38) or poorly differentiated (*n* = 26), together comprising 88.9% of the cohort. More than half of the patients presented with advanced primary tumor stage (pT3–4; 56.1%) and the majority had regional lymph node involvement (pN-positive; 60.7%). Extranodal extension (ENE) was identified in 21 of 34 patients who underwent lymph node dissection. Lymphovascular invasion (LVI) was observed in 19 of 62 patients, perineural invasion (PNI) in 29 of 54 patients, and positive surgical margins were present in 11 of 59 resected tumors. Among the 41 patients with available Worse pattern of invasion (WPOI) data, 30 (73.2%) had a WPOI-1-4, whereas 11 (26.8%) had a WPOI-5. WPOI-5 reflects the most aggressive pattern of tumor invasion, characterized by tumor satellites that are separated from the main tumor mass by at least 1 mm, as well as extratumoral perineural invasion and/or extratumoral lymphovascular invasion.

**Table 1:** Patient characteristics

	Total (*N* = 73)
*Gender*	
Female	40 (54.8%)
Male	33 (45.2%)
*Age at diagnosis, yrs*	
Median	64
Range	(19–85)
*Race*	
Caucasian	50 (71.4%)
Non-Caucasian	20 (28.6%)
Unknown	3
*Smoking history*	
Never	43 (59.7%)
Ever	29 (40.3%)
Unknown	1
*Alcohol intake history*	
Never	17 (23.3%)
Occasional	45 (61.6%)
Moderate/Heavy	11 (15.1%)
*Location of Primary Site*	
Tongue/Floor of mouth	42 (57.5%)
Gingiva	13 (17.8%)
Other	18 (24.7%)
*Pathology grade*	
Well	8 (11.1%)
Moderate	38 (52.8%)
Poor	26 (36.1%)
Unknown	1
*pT*	
1–2	29 (43.9%)
3–4	37 (56.1%)
Unknown	7
*pN*	
0	22 (39.3%)
1	5 (8.9%)
2	23 (41.1%)
3	6 (10.7%)
Unknown	17
*Lymphovascular invasion*	
Not identified	43 (69.4%)
Present	19 (30.6%)
Unknown	11
*Perineural invasion*	
Not identified	25 (46.3%)
Present	29 (53.7%)
Unknown	19
*Margins of resection*	
Not identified	48 (81.4%)
Present	11 (18.6%)
Unknown	14
*ENE*	
Not identified	13 (38.2%)
Present	21 (61.8%)
Node negative or unknown	39
*WPOI score*	
1–4	30 (73.2%)
5	11 (36.8%)
Unknown	32

### PD-L1 Immunohistochemistry

PD-L1 status was assessed by immunohistochemistry in 67 cases. Of these, 65 cases (97.0%) were PD-L1 positive, while 2 cases (3.0%) were PD-L1 negative. High PD-L1 expression was observed in 37 patients (55.2%), whereas 30 patients (44.8%) demonstrated low PD-L1 expression (Table 2).

**Table 2:** Genomic characteristics

	Total (*N* = 73)
*PD-L1*	
<20	30 (44.8%)
≥20	37 (55.2%)
Unknown	6

### DNA Sequencing Mutations

All tumors were microsatellite stable by DNA sequencing, and no clinically relevant gene fusions were identified by RNA sequencing. Most patients exhibited a low tumor mutational burden (TMB; *n* = 53), followed by intermediate TMB (*n* = 15). Only three cases demonstrated a high TMB, and TMB status could not be determined in two patients.

**Fig. 1:**
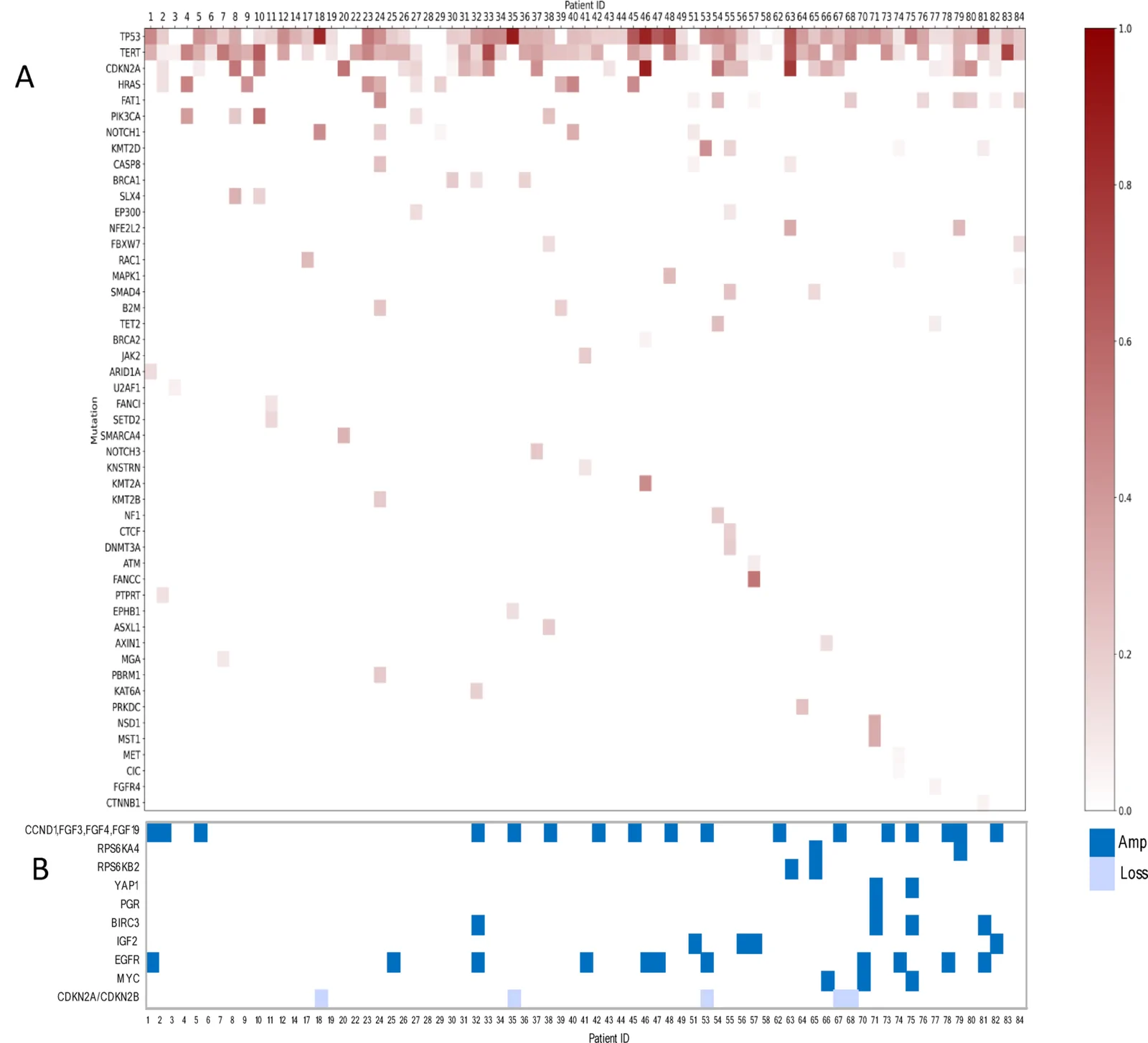
Molecular Profiling in Oral Cavity Squamous Cell Carcinoma.  **A** Heatmap plot of all genes with pathogenic variants. The heatmap contains columns which represent an individual participant (*N* = 73), and rows that represent genes, while the color intensity that fills in the cell represents the allele frequency (AF: 0–1) for a specific participant in a specific gene.** B** Heatmap plot of chromosome CNV. The copy number amplification or loss represented by the highest or lowest color intensity, respectively

The comprehensive DNA sequencing landscape of the cohort, including pathogenic and likely pathogenic somatic mutations and CNVs, is summarized in the heatmap (Fig. 1A and B).

*TP53* was the most frequently altered gene, with mutations identified in 62 patients (84.9%). The second most common alteration involved *TERT* promoter mutations, detected in 55 patients (75.3%). These included the c.-124, c.-146, and c.-57 variants, which were mutually exclusive. Other recurrent oncogenic alterations were observed in *CDKN2A*,* HRAS*,* FAT1*,* PIK3CA*,* NOTCH1*,* KMT2D*,* CASP8*,* BRCA1*,* and SLX4.*

The most frequent CNV event was amplification of chromosomal region 11q13, identified in 19 of 73 patients. This region includes *CCND1*,* FGF3*,* FGF4*, and *FGF19*, with amplification primarily localized to 11q13.3 in 17 cases (Fig. 1B). Notably, all tumors harboring 11q13 amplification also carried concurrent *TP53* mutations. Similarly, *EGFR* amplification was exclusively associated with *TP53*-mutant tumors and was present in all 11 such cases (100%). The 11q copy number amplification was mutually exclusive with *NOTCH1* mutations, which were observed in five patients.

Based on *TP53* and *TERT* promoter mutation status, the 73 cases were stratified into four molecular subgroups: *TP53*-mutant only, *TERT* promoter–mutant only, co-mutated (TP53⁺/TERT promoter⁺), and wild-type for both alterations *(TP53⁻/TERT* promoter⁻). The co-mutated subgroup demonstrated a distinct molecular profile characterized by frequent co-alterations in *CDKN2A* (*n* = 20), *FAT1* (*n* = 8), and *HRAS* (*n* = 5). CNV analysis revealed that partial 11q amplification was the most common event in this subgroup (*n* = 16), followed by *EGFR* amplification (*n* = 8). Tumors without TP53 and TERT promoter mutations typically also harbor CDKN2A alterations, including both mutations and copy number loss. Collectively, these findings reveal distinct patterns of co-occurring genomic alterations across molecular subgroups defined by *TP53* and *TERT* promoter status (Table 3 and Supplementary Fig. 1).

**Table 3:** Genomic characteristics

	Total (N=73)
*Mutations*	
*TP53* mutation	
Negative	11 (15.1%)
Single mutation	42 (57.5%)
Double/Triple mutations	20 (27.4%)
*TERT PMs*	
Negative	18 (24.7%)
Positive	55 (75.3%)
*TP53* and *TERT PMs*	
*TP53-/TERT PMs*-	3 (4.1%)
*TP53-/TERT PMs*+	8 (11%)
*TP53* single mutation/*TERT PMs*-	9 (12.3%)
*TP53* single mutation/*TERT PMs* +	33 (45.2%)
*TP53* Double/Triple mutations/*TERT PMs*-	6 (8.2%)
*TP53* Double/Triple mutations/*TERT PMs*+	14 (19.2%)
CDKN2A	
Negative	46 (63.9%)
Positive	26 (36.1%)
Unknown	1
HRAS	
Negative	63 (86.3%)
Positive	10 (13.7%)
PIK3CA	
Negative	68 (93.2%)
Positive	5 (6.8%)
*Tumor mutational burden*	
TMB score	
Low	53 (74.6%)
Intermediate	15 (21.1%)
High	3 (4.2%)
Unknown	2
*Copy number variation*	
Chr11q amp	
Not identified	52 (71.2%)
Present	21 (28.8%)
Chr9p Loss CNDK2A/B	
Not identified	68 (93.2%)
Present	5 (6.8%)
Chr7p amp (EGFR)	
Not identified	62 (84.9%)
Present	11 (15.1%)
*TP53 and Chr11q13.3 Amp*	
Negative/Not identified	11 (15.1%)
Positive/Not identified	44 (60.3%)
Positive/Present	18 (24.7%)

### TP53 Mutation Status and p53 IHC Expression Patterns

**Fig. 2:**
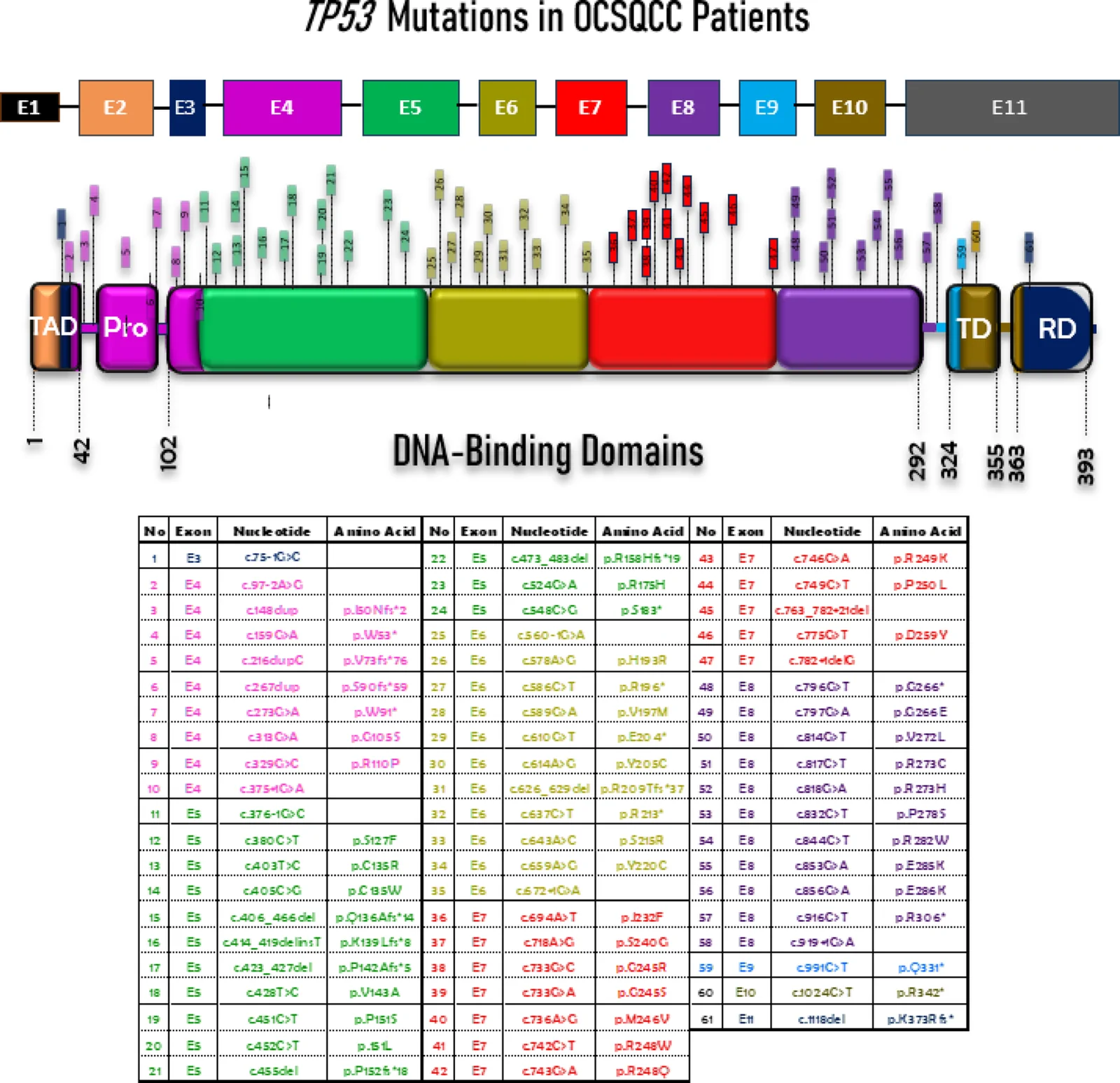
*TP53* oncogenic variants and associated IHC

A total of 62 patients harbored *TP53* alterations, predominantly affecting exons 4 through 8 (Fig. 2).

Multiple *TP53* mutations were identified in 20 of these patients. Immunohistochemistry (IHC) data were available for 46 cases; of these, 40 cases (87.0%) demonstrated aberrant *TP53* expression, manifested as either protein overexpression or complete loss of staining, whereas 6 cases (13.0%) exhibited a wild-type staining pattern.

### Recurrence versus Primary Multigene Sequencing Results

**Fig. 3:**
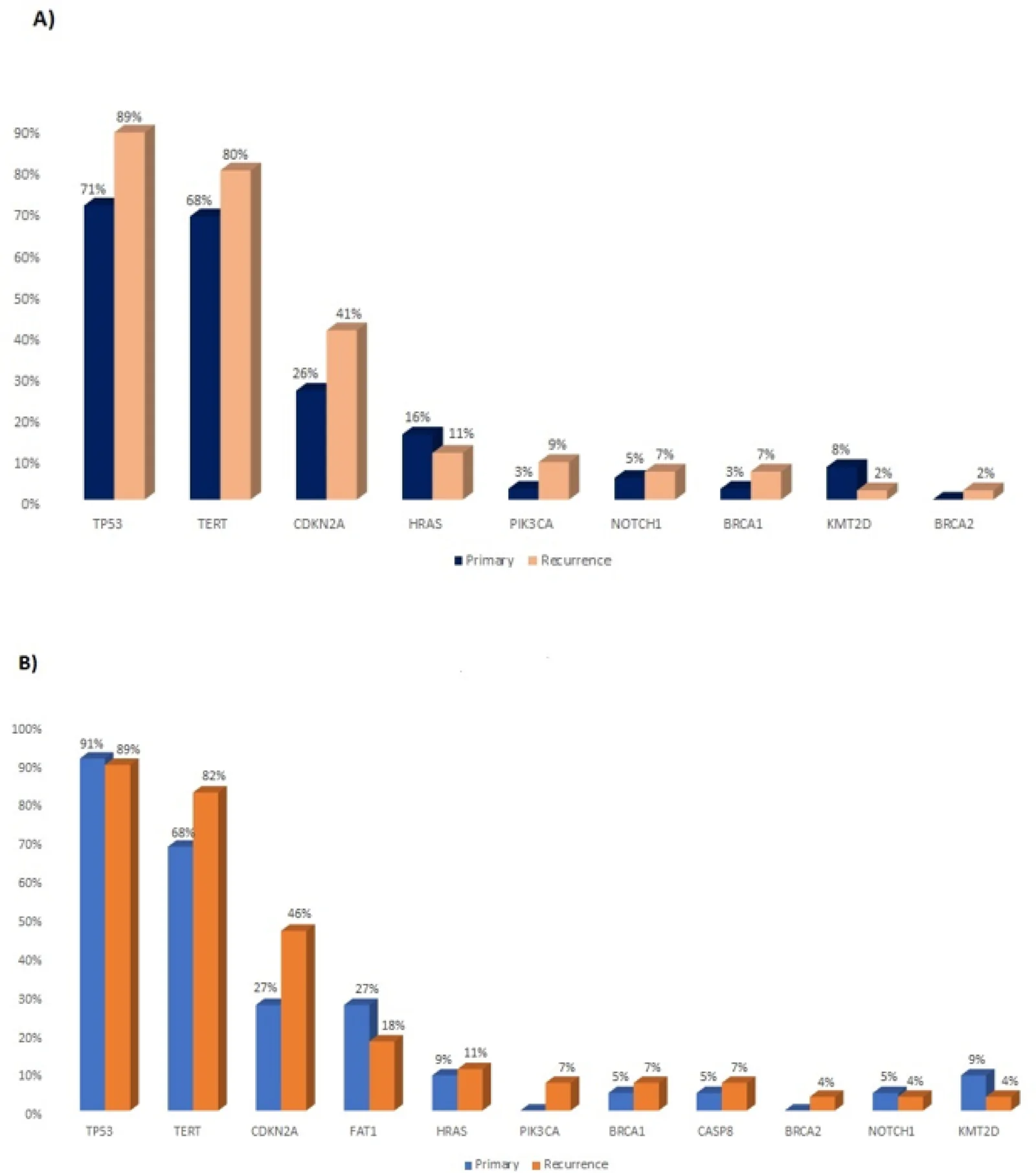
The figures illustrate the mutational profile in primary versus recurrent Disease **A** across all panels. **B** Cases evaluated using the comprehensive panel

A comparison of the genes common to all panels for primary specimens versus recurrent specimens in Fig. 3A, while the subset of cases evaluated predominantly with the comprehensive panel is shown in Fig. 3B.

### Cohort Characteristics and Survival Outcome

With a median follow-up of 45.5 months (95% CI, 35.3–59.3), the cohort demonstrated a median overall survival (OS) of 44.4 months (95% CI, 28.2–60.9) and a median disease-free survival (DFS) of 11.8 months (95% CI, 9.7–13.9).

On univariate analysis, established clinicopathologic factors associated with reduced DFS and/or OS included a history of tobacco use, advanced pathological stage (pT3–4), and regional lymph node metastasis. Positive surgical margins were significantly associated with worse OS (log-rank *p* = 0.009). Perineural invasion demonstrated borderline associations with both OS (log-rank *p* = 0.065) and DFS (log-rank *p* = 0.12), while primary tumors arising in the oral tongue or floor of mouth also showed borderline associations with OS (log-rank *p* = 0.12) and DFS (log-rank *p* = 0.19). Interestingly, younger age (< 40 years) was associated with poorer OS (log-rank *p* = 0.030) and demonstrated a borderline association with reduced DFS (log-rank *p* = 0.13).

**Fig. 4:**
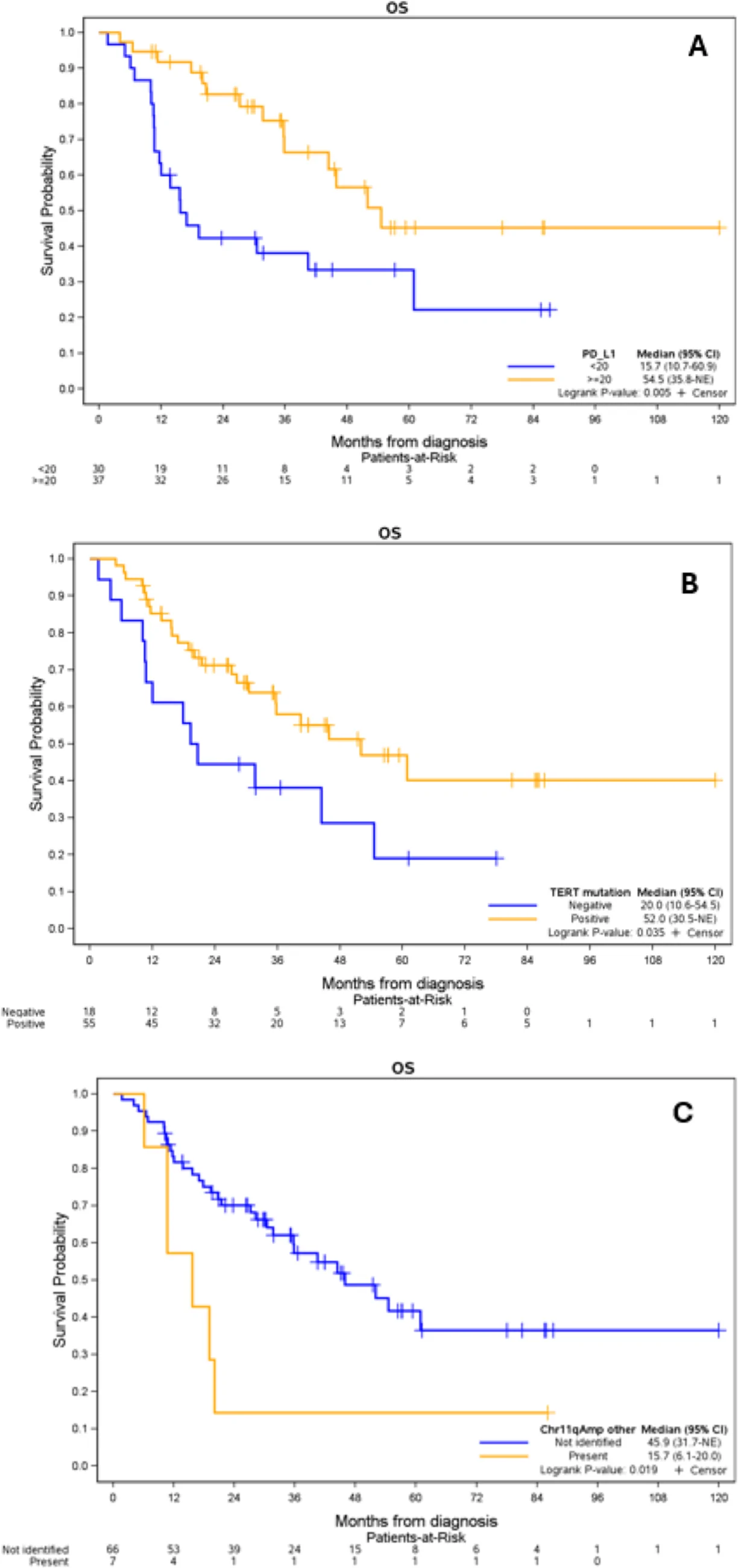
**A**,** B**, and** C**. OS association with PD-L1 IHC expression, *TERT-PMs*, and Chr11q amplification

Among the molecular variables evaluated, PD-L1 expression with a combined positive score (CPS) < 20 (log-rank *p* = 0.005), absence of *TERT*-PMs (log-rank *p* = 0.035), and focal 11q13 amplification (log-rank *p* = 0.019) were significantly associated with worse OS (Fig. 4).

In contrast, gender, race, alcohol intake, pathological grade, ENE, and LVI did not show any statistically significant association with DFS or OS. (Supplementary Fig. 2).

## Discussion

OCSCC remains a relatively poorly characterized entity, in part because of its biological heterogeneity and because it is not commonly studied independently from the other HNSCCs. In advanced or metastatic settings, OCSCC may also be misclassified as squamous cell carcinoma from another anatomic site or categorized as carcinoma of unknown primary. A limitation of prior studies is the lack of comprehensive correlation between genomic alterations and clinical outcomes, particularly with respect to tumor evolution from primary to recurrent or metastatic disease. This single-institution retrospective cohort study with longitudinal outcome follow-up aims to address these gaps by identifying surrogate biomarkers that may aid in the diagnosis and prognostication of OCSCC across different clinical settings. Furthermore, recognizing that multigene sequencing is not universally available, particularly in community practice, therefore the utility of single-gene and individual immunohistochemistry for predicting clinical outcomes in primary and recurrent OCSCC was evaluated.

Our data demonstrates that *TP53* and *TERT*-PMs are the most common alterations in OCSCC and frequently co-occur, consistent with prior studies [10, 11]. Although *TERT-*PMs have been described in a variety of solid tumors, they are particularly enriched in OCSCC among HNSCCs Moreover, the co-occurrence of *TP53* and *TERT* promoter mutations is relatively uncommon in squamous cell carcinomas of the lung and other upper aerodigestive tract sites, suggesting that this molecular signature may aid in distinguishing OCSCC from other anatomically related squamous cell carcinomas in the appropriate clinical context [7, 12–17]. Previous studies have reported conflicting results regarding the prognostic significance of *TERT*-PMs, likely reflecting heterogeneous patient cohorts, inclusion of multiple head and neck subsites, and limited site-specific analyses [11, 18–20]. In contrast, our study demonstrated significantly improved overall survival among patients harboring *TERT*-PMs, supporting their potential prognostic value in OCSCC. The high prevalence and consistent co-occurrence of *TP53* and *TERT*-PMs further suggest that these alterations represent early molecular events in OCSCC tumorigenesis and support their utility as surrogate diagnostic biomarkers in this disease.

This notion is further reinforced by recent data demonstrating *TERT*-PMs in 100% of oral proliferative verrucous leukoplakia lesions, with *TP53* mutations present in 80%, suggesting their involvement early in oral carcinogenesis [21]. Collectively, these findings highlight their diagnostic relevance. In addition, our study confirms that p53 immunohistochemistry correlates well with *TP53* mutation status and may serve as a practical alternative in settings where comprehensive genomic testing is not available.

Previous studies have associated *CASP8* mutations with adverse clinical outcomes, while the prognostic significance of *FAT1* mutations remains controversial, with conflicting reports across HNSCC cohorts [2, 22] In our study, however, neither *CASP8* nor *FAT1* alterations differed significantly between primary and recurrent tumors.

Importantly, no clinically significant RNA fusions were identified in our cohort, underscoring the limited value of RNA fusion testing in the routine genomic workup for OCSCC.

Of note previous studies in OCSCC have commonly used the standard 12 and 24-month (2-year) mark as the standard landmark for DFS evaluation and as a prognostic indicator [23, 24], while in our cohort, highest recurrence rate happened at 18 months.

Our findings identified that 11q amplification and 9p copy number loss occurred more frequently in cases with recurrence compared to those without. The *CDKN2A/B* deletion may represent a potential target for *MTAP* inhibitors, given the genomic proximity and co-deletion of *MTAP* with *CDKN2A* on chromosome 9p. Notably, *MTAP* may represent an emerging therapeutic vulnerability, as tumor cells with *MTAP* loss become dependent on *PRMT5* (protein arginine methyltransferase 5) and *MAT2A* (methionine adenosyl transferase 2 A) for survival. Ongoing clinical trials targeting these pathways are currently underway [25–27].

Copy number alterations such as amplification of chromosome 11q broadly shared across all HNSCCs [28–30], encompassing genes including *CCND1*,* FGF3*,* FGF4*, and *FGF19*, were also common. Clinically, CNV profiling emerged as one of the most informative components of genomic testing in this cohort. Amplifications involving 11q and *EGFR*, as well as deletions of *CDKN2A/B* and *MTAP*, showed meaningful associations with recurrence or survival and should be considered essential elements of OCSCC molecular evaluation.

Our cohort of patients aligns with published data indicating that smoking history, advanced tumor stage (pT3-4), positive regional lymphadenopathy, perineural invasion, and positive resection margins are all associated with worse OS and DFS [31]. Surprisingly, although ENE is a well-established adverse prognostic factor in HNSCC [32], our site-specific analysis of oral cavity cases did not demonstrate a significant association between ENE and decreased survival. This observation may be limited by the small sample size and relatively short follow-up period for some patients. However, interestingly, genomic analysis revealed that cases with ENE harbored 11q amplifications, which were strongly associated with more recurrence of OCSCC.

PD-L1 has been investigated as a predictive biomarker for response to immune checkpoint inhibitors in HNSCC; however, its role in OCSCC remains incompletely defined. Reported PD-L1 expression in OCSCC/HNSCC is highly variable, likely reflecting biologic and technical factors, including intratumoral heterogeneity, temporal changes after therapy, differences between primary and metastatic/recurrent sites, and variability in IHC antibody clones, scoring systems, and cutoffs. These factors may contribute to the conflicting prognostic and predictive associations reported across studies [9, 24, 33–35]. A prior study reported that high expression of 22C3 and other PD-L1 clones was associated with poorer OS in advanced oral SCC of the tongue and floor of the mouth, but not in other oral cavity subsites [36]. In contrast, although the tongue and floor of the mouth comprised the predominant primary sites in our cohort, we did not observe a detrimental prognostic effect of high PD-L1 expression. Instead, our data indicate that lower PD-L1 expression (CPS < 20) was associated with worse OS, suggesting a potentially distinct biological or clinical behavior within this population. Hanna et al. reported improved survival in young women with high PD-L1 expression [31], in our cohort this association was similarly observed regardless of age, including among older female patients.

An important strength of this study is its focus on location-specific molecular profiling in OCSCC, an area where data remain limited despite growing recognition of the biological heterogeneity across head and neck subsites. By assembling one of the largest cohorts to date with detailed molecular characterization and treatment-response biomarkers specific to the oral cavity, our analysis provides important granularity to the existing literature. In addition, the tertiary referral setting of our institution enabled the inclusion of clinically complex cases, offering real-world insight into patterns of advanced and recurrent disease. However, this setting also represents a limitation, as referral patterns to a tertiary cancer center may bias the study population toward more advanced disease, introducing potential selection bias.

Other limitations of this study include its retrospective design, which introduces inherent challenges such as potential confounding factors and limited control over variations in diagnostic and therapeutic practices. To enhance the reliability of our findings, we incorporated longitudinal follow-up with a median duration of 45.5 months, allowing for a more robust assessment of OS and DFS. Molecular characterization was also constrained by the inconsistent availability of tissue from both primary and metastatic lesions across patients. Additionally, the evolution of multigene NGS platforms during the study period introduced variability in genomic profiling; to mitigate this, our analyses were restricted to genes shared across the different assays (See supplementary Table 1 for details). Additionally, the absence of resection specimens in a subset of cases precluded comprehensive evaluation of several critical pathologic staging features, such as ENE and WPOI. While these limitations are intrinsic to retrospective investigations, they underscore both the complexity of studying real-world patient populations and the need for continued prospective validation to further refine pathologic and molecular risk stratification. Moreover, a more comprehensive assessment of definitive HPV status could not be established, and evaluation relied primarily on p16 immunohistochemistry as the available surrogate marker. Finally, the relatively limited sample size may constrain the power to detect some clinically meaningful associations. Despite these limitations, we hope our findings offer a meaningful step forward in unraveling the site-specific biology of OCSCC, shedding new light on its molecular complexity and helping to bridge critical gaps in our current understanding.

## Conclusion

By comparing the multigene NGS assay utility in primary versus recurrence OCSCC and separating OCSCC within a single-institution cohort, which mainly follow uniform treatment plan for patients, our study provides an understanding of the genomic events that define this disease as well as evaluating benefit of comprehensive DNA and RNA sequencing in this entity.

We identified *TP53* and *TERT*-promoter mutations as the two most frequent and co-occurring alterations in OCSCC. Although neither was associated with recurrence or overall survival in our cohort, their high prevalence and relative enrichment compared with other HNSCC subsites, specifically the HPV positive localized head and neck squamous cell carcinoma or lung squamous carcinoma suggest that they may serve as useful surrogate diagnostic biomarkers in metastatic or unknown primary tumor settings in upper aerodigestive and thoracic region. Our findings support a model in which *TP53* and *TERT*-PMs act as early cooperating events in OCSCC tumorigenesis, whereas 11q amplification, appears later in course of disease particularly gene copy number amplification involving *CCND1*,* FGF3*,* FGF4*, and *FGF1*9, contributes to disease recurrence and aggressive clinical behavior.

In addition to these diagnostic implications, our study identified several prognostic biomarkers associated with adverse outcomes, including low PD-L1 expression (< 20 CPS), absence of *TERT*-promoter mutations (with respect to OS), and 11q amplification. These markers may improve risk stratification and guide personalized treatment strategies in OCSCC.

Based on our findings, we recommend a minimum biomarker evaluation for OCSCC that includes:

1. *TP53 IHC* and *TP53* mutations testing.
2. PD-L1 immunohistochemistry.
3. No RNA sequencing for fusion detection, as currently this provides limited additional value for the genomic characterization.

Such an approach balances diagnostic and prognostic utility with accessibility, making it practical for both tertiary centers and community practices by providing a more cost-effective workup. Moreover, p53 immunohistochemistry showed strong concordance with TP53 mutation status, supporting its use as a cost-effective surrogate when comprehensive genomic profiling is not feasible.

Finally, the emergence of *TP53*-targeted therapeutic vaccines presents an exciting opportunity to translate molecular insights into preventive or therapeutic interventions, underscoring the need for prospective, multi-institutional studies to validate these findings and further refine biomarker-driven management of OCSCC [27, 37].

Looking ahead, a particularly promising avenue of investigation lies in exploring 11q amplification, or, at a minimum, CCND1 gene copy number amplification status, alongside the role of TERT promoter mutations. These molecular features may further illuminate the biological underpinnings of disease progression and behavior. In parallel, expanded transcriptomic profiling studies with larger, more diverse cohorts could unlock deeper insights from RNA sequencing data, revealing novel patterns and pathways that remain beyond the reach of current analyses.

## Supplementary Information

Below is the link to the electronic supplementary material.

Supplementary Material 1

## Data Availability

This study was conducted under the approve Intuitional board review IRB #: 15198. All data supporting the findings of this study are available within the division of Molecular Pathology and Therapy biomarkers at City of Hope Medical Centers and presented in the tables and figures of this paper and its Supplementary tables and figures provided. Raw data are available to be shared upon request.
