# Supplementary material for: An Accessible Approach to Molecular and Biomarker Testing for Risk Stratification in Oral Cavity Squamous Cell Carcinoma
Source: Head Neck Pathol. 2026 Sep 18;20(1):97. doi: 10.1007/s12105-026-01968-2 (PMC13589004; doi:10.1007/s12105-026-01968-2)
Supplement: Supplementary file 1 [file 12105_2026_1968_MOESM1_ESM.docx]

**Supplemental Table 1a.** Gene list for HopeSeq Solid Tumor Comprehensive (HSC) Testing

| **DNA Sequencing of 523 Genes, full gene (Entire Exon)** | | | | | | |
| --- | --- | --- | --- | --- | --- | --- |
| *ABL1* | *ABL2* | *ACVR1* | *ACVR1B* | *AKT1* | *AKT2* | *AKT3* |
| *ALK* | *ALOX12B* | *ANKRD11* | *ANKRD26* | *APC* | *AR* | *ARAF* |
| *ARFRP1* | *ARID1A* | *ARID1B* | *ARID2* | *ARID5B* | *ASXL1* | *ASXL2* |
| *ATM* | *ATR* | *ATRX* | *AURKA* | *AURKB* | *AXIN1* | *AXIN2* |
| *AXL* | *B2M* | *BAP1* | *BARD1* | *BBC3* | *BCL10* | *BCL2* |
| *BCL2L1* | *BCL2L11* | *BCL2L2* | *BCL6* | *BCOR* | *BCORL1* | *BCR* |
| *BIRC3* | *BLM* | *BMPR1A* | *BRAF* | *BRCA1* | *BRCA2* | *BRD4* |
| *BRIP1* | *BTG1* | *BTK* | *C11orf30* | *CALR* | *CARD11* | *CASP8* |
| *CBFB* | *CBL* | *CCND1* | *CCND2* | *CCND3* | *CCNE1* | *CD274* |
| *CD276* | *CD74* | *CD79A* | *CD79B* | *CDC73* | *CDH1* | *CDK12* |
| *CDK4* | *CDK6* | *CDK8* | *CDKN1A* | *CDKN1B* | *CDKN2A* | *CDKN2B* |
| *CDKN2C* | *CEBPA* | *CENPA* | *CHD2* | *CHD4* | *CHEK1* | *CHEK2* |
| *CIC* | *CREBBP* | *CRKL* | *CRLF2* | *CSF1R* | *CSF3R* | *CSNK1A1* |
| *CTCF* | *CTLA4* | *CTNNA1* | *CTNNB1* | *CUL3* | *CUX1* | *CXCR4* |
| *CYLD* | *DAXX* | *DCUN1D1* | *DDR2* | *DDX41* | *DHX15* | *DICER1* |
| *DIS3* | *DNAJB1* | *DNMT1* | *DNMT3A* | *DNMT3B* | *DOT1L* | *E2F3* |
| *EED* | *EGFL7* | *EGFR* | *EIF1AX* | *EIF4A2* | *EIF4E* | *EML4* |
| *EP300* | *EPCAM* | *EPHA3* | *EPHA5* | *EPHA7* | *EPHB1* | *ERBB2* |
| *ERBB3* | *ERBB4* | *ERCC1* | *ERCC2* | *ERCC3* | *ERCC4* | *ERCC5* |
| *ERG* | *ERRFI1* | *ESR1* | *ETS1* | *ETV1* | *ETV4* | *ETV5* |
| *ETV6* | *EWSR1* | *EZH2* | *FAM123B* | *FAM175A* | *FAM46C* | *FANCA* |
| *FANCC* | *FANCD2* | *FANCE* | *FANCF* | *FANCG* | *FANCI* | *FANCL* |
| *FAS* | *FAT1* | *FBXW7* | *FGF1* | *FGF10* | *FGF14* | *FGF19* |
| *FGF2* | *FGF23* | *FGF3* | *FGF4* | *FGF5* | *FGF6* | *FGF7* |
| *FGF8* | *FGF9* | *FGFR1* | *FGFR2* | *FGFR3* | *FGFR4* | *FH* |
| *FLCN* | *FLI1* | *FLT1* | *FLT3* | *FLT4* | *FOXA1* | *FOXL2* |
| *FOXO1* | *FOXP1* | *FRS2* | *FUBP1* | *FYN* | *GABRA6* | *GATA1* |
| *GATA2* | *GATA3* | *GATA4* | *GATA6* | *GEN1* | *GID4* | *GLI1* |
| *GNA11* | *GNA13* | *GNAQ* | *GNAS* | *GPR124* | *GPS2* | *GREM1* |
| *GRIN2A* | *GRM3* | *GSK3B* | *H3F3A* | *H3F3B* | *H3F3C* | *HGF* |
| *HIST1H1C* | *HIST1H2BD* | *HIST1H3A* | *HIST1H3B* | *HIST1H3C* | *HIST1H3D* | *HIST1H3E* |
| *HIST1H3F* | *HIST1H3G* | *HIST1H3H* | *HIST1H3I* | *HIST1H3J* | *HIST2H3A* | *HIST2H3C* |
| *HIST2H3D* | *HIST3H3* | *HLA-A* | *HLA-B* | *HLA-C* | *HNF1A* | *HNRNPK* |
| *HOXB13* | *HRAS* | *HSD3B1* | *HSP90AA1* | *ICOSLG* | *ID3* | *IDH1* |
| *IDH2* | *IFNGR1* | *IGF1* | *IGF1R* | *IGF2* | *IKBKE* | *IKZF1* |
| *IL10* | *IL7R* | *INHA* | *INHBA* | *INPP4A* | *INPP4B* | *INSR* |
| *IRF2* | *IRF4* | *IRS1* | *IRS2* | *JAK1* | *JAK2* | *JAK3* |
| *JUN* | *KAT6A* | *KDM5A* | *KDM5C* | *KDM6A* | *KDR* | *KEAP1* |
| *KEL* | *KIF5B* | *KIT* | *KLF4* | *KLHL6* | *KMT2B* | *KMT2C* |
| *KMT2D* | *KRAS* | *LAMP1* | *LATS1* | *LATS2* | *LMO1* | *LRP1B* |
| *LYN* | *LZTR1* | *MAGI2* | *MALT1* | *MAP2K1* | *MAP2K2* | *MAP2K4* |
| *MAP3K1* | *MAP3K13* | *MAP3K14* | *MAP3K4* | *MAPK1* | *MAPK3* | *MAX* |
| *MCL1* | *MDC1* | *MDM2* | *MDM4* | *MED12* | *MEF2B* | *MEN1* |
| *MET* | *MGA* | *MITF* | *MLH1* | *MLL* | *MLLT3* | *MPL* |
| *MRE11A* | *MSH2* | *MSH3* | *MSH6* | *MST1* | *MST1R* | *MTOR* |
| *MUTYH* | *MYB* | *MYC* | *MYCL1* | *MYCN* | *MYD88* | *MYOD1* |
| *NAB2* | *NBN* | *NCOA3* | *NCOR1* | *NEGR1* | *NF1* | *NF2* |
| *NFE2L2* | *NFKBIA* | *NKX2-1* | *NKX3-1* | *NOTCH1* | *NOTCH2* | *NOTCH3* |
| *NOTCH4* | *NPM1* | *NRAS* | *NRG1* | *NSD1* | *NTRK1* | *NTRK2* |
| *NTRK3* | *NUP93* | *NUTM1* | *PAK1* | *PAK3* | *PAK7* | *PALB2* |
| *PARK2* | *PARP1* | *PAX3* | *PAX5* | *PAX7* | *PAX8* | *PBRM1* |
| *PDCD1* | *PDCD1LG2* | *PDGFRA* | *PDGFRB* | *PDK1* | *PDPK1* | *PGR* |
| *PHF6* | *PHOX2B* | *PIK3C2B* | *PIK3C2G* | *PIK3C3* | *PIK3CA* | *PIK3CB* |
| *PIK3CD* | *PIK3CG* | *PIK3R1* | *PIK3R2* | *PIK3R3* | *PIM1* | *PLCG2* |
| *PLK2* | *PMAIP1* | *PMS1* | *PMS2* | *PNRC1* | *POLD1* | *POLE* |
| *PPARG* | *PPM1D* | *PPP2R1A* | *PPP2R2A* | *PPP6C* | *PRDM1* | *PREX2* |
| *PRKAR1A* | *PRKCI* | *PRKDC* | *PRSS8* | *PTCH1* | *PTEN* | *PTPN11* |
| *PTPRD* | *PTPRS* | *PTPRT* | *QKI* | *RAB35* | *RAC1* | *RAD21* |
| *RAD50* | *RAD51* | *RAD51B* | *RAD51C* | *RAD51D* | *RAD52* | *RAD54L* |
| *RAF1* | *RANBP2* | *RARA* | *RASA1* | *RB1* | *RBM10* | *RECQL4* |
| *REL* | *RET* | *RFWD2* | *RHEB* | *RHOA* | *RICTOR* | *RIT1* |
| *RNF43* | *ROS1* | *RPS6KA4* | *RPS6KB1* | *RPS6KB2* | *RPTOR* | *RUNX1* |
| *RUNX1T1* | *RYBP* | *SDHA* | *SDHAF2* | *SDHB* | *SDHC* | *SDHD* |
| *SETBP1* | *SETD2* | *SF3B1* | *SH2B3* | *SH2D1A* | *SHQ1* | *SLIT2* |
| *SLX4* | *SMAD2* | *SMAD3* | *SMAD4* | *SMARCA4* | *SMARCB1* | *SMARCD1* |
| *SMC1A* | *SMC3* | *SMO* | *SNCAIP* | *SOCS1* | *SOX10* | *SOX17* |
| *SOX2* | *SOX9* | *SPEN* | *SPOP* | *SPTA1* | *SRC* | *SRSF2* |
| *STAG1* | *STAG2* | *STAT3* | *STAT4* | *STAT5A* | *STAT5B* | *STK11* |
| *STK40* | *SUFU* | *SUZ12* | *SYK* | *TAF1* | *TBX3* | *TCEB1* |
| *TCF3* | *TCF7L2* | *TERC* | *TERT* | *TET1* | *TET2* | *TFE3* |
| *TFRC* | *TGFBR1* | *TGFBR2* | *TMEM127* | *TMPRSS2* | *TNFAIP3* | *TNFRSF14* |
| *TOP1* | *TOP2A* | *TP53* | *TP63* | *TRAF2* | *TRAF7* | *TSC1* |
| *TSC2* | *TSHR* | *U2AF1* | *VEGFA* | *VHL* | *VTCN1* | *WISP3* |
| *WT1* | *XIAP* | *XPO1* | *XRCC2* | *YAP1* | *YES1* | *ZBTB2* |
| *ZBTB7A* | *ZFHX3* | *ZNF217* | *ZNF703* | *ZRSR2* |  |  |
|  |  |  |  |  |  |  |
| **Copy Number Alterations of 500 Genes** | | | | | | |
| *ABL1* | *ABL2* | *ACVR1* | *ACVR1B* | *AKT1* | *AKT2* | *AKT3* |
| *ALK* | *ALOX12B* | *ANKRD11* | *ANKRD26* | *APC* | *AR* | *ARAF* |
| *ARFRP1* | *ARID1A* | *ARID1B* | *ARID2* | *ARID5B* | *ASXL1* | *ASXL2* |
| *ATM* | *ATR* | *ATRX* | *AURKA* | *AURKB* | *AXIN1* | *AXIN2* |
| *AXL* | *B2M* | *BAP1* | *BARD1* | *BBC3* | *BCL10* | *BCL2* |
| *BCL2L1* | *BCL2L11* | *BCL2L2* | *BCL6* | *BCOR* | *BCORL1* | *BCR* |
| *BIRC3* | *BLM* | *BMPR1A* | *BRAF* | *BRCA1* | *BRCA2* | *BRD4* |
| *BRIP1* | *BTG1* | *BTK* | *C11orf30* | *CALR* | *CARD11* | *CASP8* |
| *CBFB* | *CBL* | *CCND1* | *CCND2* | *CCND3* | *CCNE1* | *CD274* |
| *CD276* | *CD74* | *CD79A* | *CD79B* | *CDC73* | *CDH1* | *CDK12* |
| *CDK4* | *CDK6* | *CDK8* | *CDKN1A* | *CDKN1B* | *CDKN2A* | *CDKN2B* |
| *CDKN2C* | *CEBPA* | *CENPA* | *CHD2* | *CHD4* | *CHEK1* | *CHEK2* |
| *CIC* | *CREBBP* | *CRKL* | *CRLF2* | *CSF1R* | *CSF3R* | *CSNK1A1* |
| *CTCF* | *CTLA4* | *CTNNA1* | *CTNNB1* | *CUL3* | *CUX1* | *CXCR4* |
| *CYLD* | *DAXX* | *DCUN1D1* | *DDR2* | *DDX41* | *DHX15* | *DICER1* |
| *DIS3* | *DNMT1* | *DNMT3A* | *DNMT3B* | *DOT1L* | *E2F3* | *EED* |
| *EGFL7* | *EGFR* | *EIF1AX* | *EIF4A2* | *EIF4E* | *EML4* | *EP300* |
| *EPCAM* | *EPHA3* | *EPHA5* | *EPHA7* | *EPHB1* | *ERBB2* | *ERBB3* |
| *ERBB4* | *ERCC1* | *ERCC2* | *ERCC3* | *ERCC4* | *ERCC5* | *ERG* |
| *ERRFI1* | *ESR1* | *ETS1* | *ETV1* | *ETV4* | *ETV5* | *ETV6* |
| *EWSR1* | *EZH2* | *FAM123B* | *FAM175A* | *FAM46C* | *FANCA* | *FANCC* |
| *FANCD2* | *FANCE* | *FANCG* | *FANCI* | *FANCL* | *FAS* | *FAT1* |
| *FBXW7* | *FGF1* | *FGF10* | *FGF14* | *FGF19* | *FGF2* | *FGF23* |
| *FGF3* | *FGF4* | *FGF5* | *FGF6* | *FGF7* | *FGF8* | *FGF9* |
| *FGFR1* | *FGFR2* | *FGFR3* | *FGFR4* | *FH* | *FLCN* | *FLI1* |
| *FLT1* | *FLT3* | *FLT4* | *FOXA1* | *FOXO1* | *FOXP1* | *FRS2* |
| *FUBP1* | *FYN* | *GABRA6* | *GATA1* | *GATA2* | *GATA3* | *GATA4* |
| *GATA6* | *GEN1* | *GID4* | *GLI1* | *GNA11* | *GNA13* | *GNAQ* |
| *GNAS* | *GPR124* | *GPS2* | *GREM1* | *GRIN2A* | *GRM3* | *GSK3B* |
| *H3F3A* | *H3F3B* | *H3F3C* | *HGF* | *HIST1H1C* | *HIST1H2BD* | *HIST1H3B* |
| *HIST3H3* | *HNF1A* | *HNRNPK* | *HOXB13* | *HRAS* | *HSD3B1* | *HSP90AA1* |
| *ICOSLG* | *ID3* | *IDH1* | *IDH2* | *IFNGR1* | *IGF1* | *IGF1R* |
| *IGF2* | *IKBKE* | *IKZF1* | *IL10* | *IL7R* | *INHA* | *INHBA* |
| *INPP4A* | *INPP4B* | *INSR* | *IRF2* | *IRF4* | *IRS1* | *IRS2* |
| *JAK1* | *JAK2* | *JAK3* | *JUN* | *KAT6A* | *KDM5A* | *KDM5C* |
| *KDM6A* | *KDR* | *KEAP1* | *KEL* | *KIF5B* | *KIT* | *KLF4* |
| *KLHL6* | *KRAS* | *LAMP1* | *LATS1* | *LATS2* | *LMO1* | *LRP1B* |
| *LYN* | *LZTR1* | *MAGI2* | *MALT1* | *MAP2K1* | *MAP2K2* | *MAP2K4* |
| *MAP3K1* | *MAP3K13* | *MAP3K14* | *MAP3K4* | *MAPK1* | *MAPK3* | *MAX* |
| *MCL1* | *MDC1* | *MDM2* | *MDM4* | *MED12* | *MEF2B* | *MEN1* |
| *MET* | *MGA* | *MITF* | *MLH1* | *MLL* | *MLLT3* | *MPL* |
| *MRE11A* | *MSH2* | *MSH3* | *MSH6* | *MST1* | *MST1R* | *MTOR* |
| *MUTYH* | *MYB* | *MYC* | *MYCL1* | *MYCN* | *MYD88* | *MYOD1* |
| *NAB2* | *NBN* | *NCOA3* | *NCOR1* | *NEGR1* | *NF1* | *NF2* |
| *NFE2L2* | *NFKBIA* | *NKX2-1* | *NKX3-1* | *NOTCH1* | *NOTCH2* | *NOTCH3* |
| *NOTCH4* | *NPM1* | *NRAS* | *NRG1* | *NSD1* | *NTRK1* | *NTRK2* |
| *NTRK3* | *NUP93* | *NUTM1* | *PAK1* | *PAK3* | *PAK7* | *PALB2* |
| *PARK2* | *PARP1* | *PAX3* | *PAX5* | *PAX7* | *PAX8* | *PBRM1* |
| *PDCD1* | *PDCD1LG2* | *PDGFRA* | *PDGFRB* | *PDK1* | *PDPK1* | *PGR* |
| *PHF6* | *PHOX2B* | *PIK3C2B* | *PIK3C2G* | *PIK3C3* | *PIK3CA* | *PIK3CB* |
| *PIK3CD* | *PIK3CG* | *PIK3R1* | *PIK3R2* | *PIK3R3* | *PIM1* | *PLCG2* |
| *PLK2* | *PMAIP1* | *PMS1* | *PMS2* | *PNRC1* | *POLD1* | *POLE* |
| *PPARG* | *PPM1D* | *PPP2R1A* | *PPP2R2A* | *PPP6C* | *PRDM1* | *PREX2* |
| *PRKAR1A* | *PRKCI* | *PRKDC* | *PRSS8* | *PTCH1* | *PTEN* | *PTPN11* |
| *PTPRD* | *PTPRS* | *PTPRT* | *QKI* | *RAB35* | *RAC1* | *RAD21* |
| *RAD50* | *RAD51* | *RAD51B* | *RAD51C* | *RAD51D* | *RAD52* | *RAD54L* |
| *RAF1* | *RANBP2* | *RARA* | *RASA1* | *RB1* | *RBM10* | *RECQL4* |
| *REL* | *RET* | *RFWD2* | *RHEB* | *RHOA* | *RICTOR* | *RIT1* |
| *RNF43* | *ROS1* | *RPS6KA4* | *RPS6KB1* | *RPS6KB2* | *RPTOR* | *RUNX1* |
| *RUNX1T1* | *RYBP* | *SDHA* | *SDHAF2* | *SDHB* | *SDHC* | *SDHD* |
| *SETBP1* | *SETD2* | *SF3B1* | *SH2B3* | *SH2D1A* | *SHQ1* | *SLIT2* |
| *SLX4* | *SMAD2* | *SMAD3* | *SMAD4* | *SMARCA4* | *SMARCB1* | *SMARCD1* |
| *SMC1A* | *SMC3* | *SMO* | *SNCAIP* | *SOCS1* | *SOX10* | *SOX17* |
| *SOX2* | *SOX9* | *SPEN* | *SPOP* | *SPTA1* | *SRC* | *SRSF2* |
| *STAG1* | *STAG2* | *STAT3* | *STAT4* | *STAT5A* | *STAT5B* | *STK11* |
| *STK40* | *SUFU* | *SUZ12* | *SYK* | *TAF1* | *TBX3* | *TCEB1* |
| *TCF3* | *TCF7L2* | *TET1* | *TET2* | *TFE3* | *TFRC* | *TGFBR1* |
| *TGFBR2* | *TMEM127* | *TMPRSS2* | *TNFAIP3* | *TNFRSF14* | *TOP1* | *TOP2A* |
| *TP53* | *TP63* | *TRAF2* | *TRAF7* | *TSC1* | *TSC2* | *TSHR* |
| *U2AF1* | *VEGFA* | *VHL* | *VTCN1* | *WISP3* | *WT1* | *XIAP* |
| *XPO1* | *XRCC2* | *YAP1* | *YES1* | *ZBTB2* | *ZBTB7A* | *ZFHX3* |
| *ZNF217* | *ZNF703* | *ZRSR2* |  |  |  |  |
|  |  |  |  |  |  |  |
| **Fusion Analysis of 165 Genes (RNA Sequencing)** | | | | | | |
| *ABL1* | *ABL2* | *AKT3* | *ALK* | *ARHGAP26* | *AXL* | *BCL11B* |
| *BCL2* | *BCL6* | *BCOR* | *BCR* | *BIRC3* | *BRAF* | *BRD3* |
| *BRD4* | *CAMTA1* | *CBFB* | *CCNB3* | *CCND1* | *CCND3* | *CD151* |
| *CDK6* | *CHD1* | *CHIC2* | *CIC* | *CIITA* | *CREBBP* | *CRLF2* |
| *CSF1R* | *DEK* | *DUSP22* | *EBF1* | *EGFR* | *EIF4A1* | *EPC1* |
| *EPOR* | *ERG* | *ESR1* | *ESRRA* | *ETV1* | *ETV4* | *ETV5* |
| *ETV6* | *EWSR1* | *FGFR1* | *FGFR2* | *FGFR3* | *FGR* | *FOSB* |
| *FOXO1* | *FUS* | *GLI1* | *GLIS2* | *HMGA2* | *IKZF1* | *IKZF2* |
| *IKZF3* | *IL2RB* | *INSR* | *JAK2* | *JAZF1* | *KAT6A* | *KLF2* |
| *KMT2A* | *LMO2* | *LYN* | *MALT1* | *MAML2* | *MAN2B1* | *MAST1* |
| *MAST2* | *MBTD1* | *MEAF6* | *MECOM* | *MEF2D* | *MET* | *MGEA5* |
| *MKL1* | *MKL2* | *MLF1* | *MLLT10* | *MLLT4* | *MN1* | *MSMB* |
| *MUSK* | *MYB* | *MYC* | *MYH11* | *NCOA1* | *NCOA2* | *NF1* |
| *NFKB2* | *NOTCH1* | *NOTCH2* | *NPM1* | *NR4A3* | *NRG1* | *NTRK1* |
| *NTRK2* | *NTRK3* | *NUMBL* | *NUP214* | *NUP98* | *NUTM1* | *P2RY8* |
| *PAG1* | *PAX3* | *PAX5* | *PAX7* | *PBX1* | *PDCD1LG2* | *PDGFB* |
| *PDGFRA* | *PDGFRB* | *PHF1* | *PICALM* | *PIK3CA* | *PKN1* | *PLAG1* |
| *PML* | *PPARG* | *PRDM16* | *PRKCA* | *PRKCB* | *PRKD1* | *PRKD2* |
| *PRKD3* | *PTK2B* | *RAF1* | *RANBP17* | *RARA* | *RBM15* | *RECK* |
| *RELA* | *RET* | *ROS1* | *RSPO2* | *RSPO3* | *RUNX1* | *RUNX1T1* |
| *SEMA6A* | *SETD2* | *SS18* | *STAG2* | *STAT6* | *STIL* | *TAF15* |
| *TAL1* | *TCF12* | *TCF3* | *TERT* | *TFE3* | *TFEB* | *TFG* |
| *THADA* | *TMPRSS2* | *TP63* | *TSLP* | *TYK2* | *USP6* | *YAP1* |
| *YWHAE* | *ZCCHC7* | *ZMYND11* | *ZNF384* |  |  |  |

**Supplementary Table 1b.** Gene list for HopeSeq Solid Tumor Complete (HC) Testing

| **Hotspot Mutation Analysis of 87 Genes** | | | | | | |
| --- | --- | --- | --- | --- | --- | --- |
| *AKT1* | *CHEK2* | *FGFR3* | *IDH2* | *MAX* | *PDGFRA* | *SMAD4* |
| *AKT2* | *CSF1R* | *FGFR4* | *JAK1* | *MDM4* | *PDGFRB* | *SMO* |
| *AKT3* | *CTNNB1* | *FLT3* | *JAK2* | *MED12* | *PIK3CA* | *SPOP* |
| *ALK* | *DDR2* | *FOXL2* | *JAK3* | *MET* | *PIK3CB* | *SRC* |
| *AR* | *EGFR* | *GATA2* | *KDR* | *MTOR* | *PPP2R1A* | *STAT3* |
| *ARAF* | *ERBB2* | *GNA11* | *KIT* | *MYC* | *PTPN11* | *TERT* |
| *AXL* | *ERBB3* | *GNAQ* | *KNSTRN* | *MYCN* | *RAC1* | *TOP1* |
| *BRAF* | *ERBB4* | *GNAS* | *KRAS* | *MYD88* | *RAF1* | *U2AF1* |
| *BTK* | *ERCC2* | *H3F3A* | *MAGOH* | *NFE2L2* | *RET* | *XPO1* |
| *CBL* | *ESR1* | *HIST1H3B* | *MAP2K1* | *NRAS* | *RHEB* |  |
| *CCND1* | *EZH2* | *HNF1A* | *MAP2K2* | *NTRK1* | *RHOA* |  |
| *CDK4* | *FGFR1* | *HRAS* | *MAP2K4* | *NTRK2* | *ROS1* |  |
| *CDK6* | *FGFR2* | *IDH1* | *MAPK1* | *NTRK3* | *SF3B1* |  |
| **Entire Coding Region Mutation Analysis of 48 Genes** | | | | | | |
| *ARID1A* | *CDK12* | *FANCD2* | *NBN* | *PIK3R1* | *RAD51B* | *SMARCA4* |
| *ATM* | *CDKN1B* | *FANCI* | *NF1* | *PMS2* | *RAD51C* | *SMARCB1* |
| *ATR* | *CDKN2A* | *FBXW7* | *NF2* | *POLE* | *RAD51D* | *STK11* |
| *ATRX* | *CDKN2B* | *MLH1* | *NOTCH1* | *PTCH1* | *RB1* | *TP53* |
| *BAP1* | *CHEK1* | *MRE11A* | *NOTCH2* | *PTEN* | *RNF43* | *TSC1* |
| *BRCA1* | *CREBBP* | *MSH2* | *NOTCH3* | *RAD50* | *SETD2* | *TSC2* |
| *BRCA2* | *FANCA* | *MSH6* | *PALB2* | *RAD51* | *SLX4* |  |
| **Fusion Analysis of 51 Genes** | | | | | | |
| *AKT2* | *EGFR* | *FGFR1* | *MET* | *NTRK2* | *PRKACB* | *RSPO2* |
| *ALK* | *ERBB2* | *FGFR2* | *MYB* | *NTRK3* | *PTEN* | *RSPO3* |
| *AR* | *ERBB4* | *FGFR3* | *MYBL1* | *NUTM1* | *RAD51B* | *TERT* |
| *AXL* | *ERG* | *FGR* | *NF1* | *PDGFRA* | *RAF1* |  |
| *BRAF* | *ESR1* | *FLT3* | *NOTCH1* | *PDGFRB* | *RB1* |  |
| *BRCA1* | *ETV1* | *JAK2* | *NOTCH4* | *PIK3CA* | *RELA* |  |
| *BRCA2* | *ETV4* | *KRAS* | *NRG1* | *PPARG* | *RET* |  |
| *CDKN2A* | *ETV5* | *MDM4* | *NTRK1* | *PRKACA* | *ROS1* |  |
| **Copy Number Analysis of 47 Genes** | | | | | | |
| *AKT1* | *CCND1* | *CDKN2A* | *FGFR1* | *KRAS* | *NTRK1* | *PPARG* |
| *AKT2* | *CCND2* | *CDKN2B* | *FGFR2* | *MDM2* | *NTRK2* | *RICTOR* |
| *AKT3* | *CCND3* | *EGFR* | *FGFR3* | *MDM4* | *NTRK3* | *TERT* |
| *ALK* | *CCNE1* | *ERBB2* | *FGFR4* | *MET* | *PDGFRA* | *TSC1* |
| *AR* | *CDK2* | *ESR1* | *FLT3* | *MYC* | *PDGFRB* | *TSC2* |
| *AXL* | *CDK4* | *FGF3* | *IGF1R* | *MYCL* | *PIK3CA* |  |
| *BRAF* | *CDK6* | *FGF19* | *KIT* | *MYCN* | *PIK3CB* |  |

**Supplementary Figure legends:**

**Supplementary Figure 1. Distribution of TP53 and TERT promoter mutations and co-mutation patterns in OCSCC.**

**(1)** Distribution of cases according to **TP53** and **TERT promoter mutation (TERT-PM)** status, categorized into five molecular groups. A pie chart illustrates the relative proportion of each molecular subgroup. **(1A)** Tumors harboring both TP53 and TERT-PMs, with co-mutations and co-copy number alterations pie charts **(1B)** Frequency of co-occurring genomic alterations in tumors harboring **TP53 mutations only**. **(1C)** Frequency of co-occurring genomic alterations in tumors harboring **TERT promoter mutations only**. **(1D)** Frequency of co-occurring genomic alterations in tumors lacking both **TP53** and **TERT promoter mutations (1E)** Table B and Table D data is merged to illustrate solely co- mutation and co-copy number alterations pattern in all cases without TERT PMs.

**(1A)** Tumors harboring both TP53 and TERT-PMs (blue Pie of the chart demonstrated in two pie charts the right above demonstrated co-mutations, the right below demonstrated co-Copy number variations

Fig-1A

**(1B)** Frequency of co-occurring genomic alterations in tumors harboring **TP53 mutations only (without TERT)**.

|  |  |
| --- | --- |
| CDKN2A | 0.188 |
| KMT2D | 0.063 |
| FAT1 | 0.063 |
| U2AF1 | 0.063 |
| FANCI | 0.063 |
| EPHB1 | 0.063 |
| TET2 | 0.063 |
| AXIN1 | 0.063 |
| NSD1 | 0.063 |
| MST1 | 0.063 |
| FGFR4 | 0.063 |
| TP63 | 0.063 |
|  | 0 |
| 11q13.3 (CCND1/FGF3/FGF/FGF9) | 0.188 |
| 11q22.1 (YAP1 ) | 0.125 |
| 11q22.1 (PGR ) | 0.063 |
| 11q22.2 (BIRC3 ) | 0.125 |
| 7p (EGFR) | 0.188 |
| 9p21.3L (CDKN2A/B) | 0.188 |
| 8q24.21 (MYC) | 0.188 |
| 5p13.1 (RICTOR) | 0.063 |
| 5p13.2 (IL7R) | 0.063 |
| 5p15.33 (TERT) | 0.063 |

|  |  |
| --- | --- |
| CDKN2A | 0.125 |
| HRAS | 0.625 |
| PIK3CA | 0.25 |
| NOTCH1 | 0.25 |
| EP300 | 0.125 |
| FAT1 | 0.125 |
| U2AF1 | 0.125 |
| CASP8 | 0.125 |
| B2M | 0.125 |
| 11p15.5 (IGF2 ) | 0.125 |

**(1C)** Frequency of co-occurring genomic alterations in tumors harboring **TERT promoter mutations only (without TP53)**.

|  |  |
| --- | --- |
| CDKN2A | 0.25 |
| HRAS | 0.25 |
| NOTCH1 | 0.25 |
| SMARCA4 | 0.25 |
| 9p21.3L (CDKN2A/B) | 0.25 |

**(1D)** Frequency of co-occurring genomic alterations in tumors Without **TP53** and **TERT promoter mutations**.

Fig-1E

**(1E) Illustrate co-mutation pattern in entire cases without TERT PM, which demonstrated CDKN2A mutations, followed by CAN of 11q13 and CNL of 9p, and CNA of EGFR and MYC are most common alterations in absence of TERT PMs.**

**Differences of this cohort with 1C is in this pie chart the TERT negative group/TP53 only (1B) is combined with Neither TP53 and TERT PMs group (1D).**

**Supplementary Figure 2. Kaplan–Meier survival analyses of clinicopathologic variables that were not significantly associated with survival outcomes.**

Kaplan–Meier curves demonstrating **overall survival (OS)** and **disease-free survival (DFS)** according to clinicopathologic and epidemiologic variables that did not reach statistical significance on univariate analysis. Variables include epidemiologic characteristics (e.g., sex, alcohol use, and race/ethnicity, where applicable) and clinicopathologic features not significantly associated with survival outcomes.

**Supplementary Figure 1. Distribution of TP53 and TERT promoter mutations and co-mutation patterns in OCSCC.**

**(A**.
